# Inhibition of IL‐6/STAT3 Signaling by N‐Trans‐Hibiscusamide and Its Derivative in a Mouse Model of Collagen‐Induced Arthritis

**DOI:** 10.1002/fsn3.71601

**Published:** 2026-03-04

**Authors:** Hyung Jin Lim, Seon Gyeong Bak, Jaehoon Bae, Nisansala Chandimali, Eun Jae Park, Seung Woong Lee, Mingyeong Kim, Sang‐Hoon Lee, Yeong‐Seon Won, Sun Hee Cheong, Seung‐Jae Lee

**Affiliations:** ^1^ Scripps Korea Antibody Institute Chuncheon Korea; ^2^ Functional Biomaterial Research Center Korea Research Institute of Bioscience and Biotechnology (KRIBB) Jeongeup Korea; ^3^ Functional Food Research Institute, Industry‐University Cooperation Foundation Daegu‐Hanny University gyeongsan Korea; ^4^ Department of Applied Biological Engineering, Biotechnology of KRIBB School University of Science and Technology Daejeon Korea; ^5^ Division of Functional Food Research Korea Food Research Institute Wanju Korea; ^6^ Department of Food Biotechnology University of Science and Technology (UST) Daejeon Korea; ^7^ Division of Research Management, Department of Bioresource Industrialization Honam National Institute of Biological Resource Mokpo Korea; ^8^ Department of Marine Bio Food Science Chonnam National University Yeosu Korea

**Keywords:** *4‐O‐(E)‐*feruloyl‐*N‐(E)‐*hibiscusamide, collagen‐induced arthritis, IL‐6/STAT3, *N‐trans*‐hibiscusamide

## Abstract

Rheumatoid arthritis is a chronic autoimmune disease characterized by persistent joint inflammation and progressive joint destruction, leading to pain, disability, and reduced quality of life. Interleukin‐6 is a key pro‐inflammatory cytokine that plays a central role in the pathogenesis of rheumatoid arthritis by activating downstream inflammatory signaling pathways. Dysregulation of the interleukin‐6‐mediated signaling cascade, particularly the signal transducer and activator of transcription 3 pathway, contributes to sustained inflammation and disease progression, highlighting this axis as an important therapeutic target. Rheumatoid arthritis (RA) is a chronic autoimmune disorder characterized by joint inflammation and destruction, with interleukin‐6 (IL‐6) playing a central role in its pathogenesis by driving inflammatory responses. Targeting the IL‐6/signal transducer and activator of transcription 3 (STAT3) pathway has emerged as a promising therapeutic approach for RA. This study investigates the inhibitory effects and underlying mechanisms of *N*‐trans‐hibiscusamide (NHA) and its derivative 4‐*O*‐(*E*)‐feruloyl‐*N*‐(*E*)‐hibiscusamide (HAD) on the IL‐6/STAT3 signaling axis. Using a phosphorylated STAT3 luciferase reporter assay, NHA and HAD significantly reduced IL‐6‐induced luciferase activity. They also downregulated IL‐6‐induced gene expression, inhibited STAT3 nuclear translocation and phosphorylation of signaling molecules, and suppressed IL‐6/interleukin‐6 receptor binding. In a collagen‐induced arthritis mouse model, both compounds alleviated arthritis symptoms, decreased serum levels of anti‐type II collagen immunoglobulin G and interleukin‐17A, and downregulated T helper 17‐specific genes in the spleen. Furthermore, in vitro experiments demonstrated that NHA and HAD inhibited the differentiation of naïve CD4‐positive T cells into T helper 17 cells. These findings suggest that NHA and HAD effectively modulate interleukin‐6–mediated inflammatory signaling and may serve as potential therapeutic candidates for the management of rheumatoid arthritis.

## Graphical abstract

The IL‐6/STAT3 axis is crucial in rheumatoid arthritis (RA) via Th17 differentiation. The IL‐6/STAT3 signaling pathway is downregulated as a result of the inhibition of IL‐6 and IL‐6R binding by NHA and NAD. NHA and NAD decrease the Th17 differentiation, which lessens the severity of CIA.

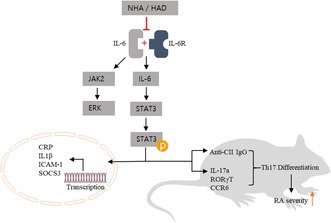

**Abbreviations**
CIA: collagen‐induced arthritis
CII: collagen II
CRP: C‐reactive protein
gp130: glycoprotein 130
HAD: 4‐*O*‐(*E*)‐feruloyl‐*N*‐(*E*)‐hibiscusamide
ICAM‐1: intercellular adhesion molecule‐1
IL‐1β: interleukin‐1 beta
IL‐6: interleukin‐6
NHA: *N*‐trans‐hibiscusamide
RORγT: retinoic acid receptor‐related orphan receptor gamma T
SOCS 3: suppressor of cytokine signaling 3

## 1 Introduction

IL‐6 is a multifunctional cytokine playing a central role in regulating inflammatory processes (Su et al. 2017). In inflammatory conditions, IL‐6 levels typically rise, binding to IL‐6R and recruiting glycoprotein 130 (gp130). These proteins assemble into the IL‐6/IL‐6R/gp130 hexamer, and gp130‐bound Janus kinase 2 (JAK2) is brought closer (Wang and Sun 2014). The adjoining JAK2 is trans‐phosphorylated, and the tyrosine residues of gp130 are phosphorylated by pJAK2 (Wang and Fuller 1994). This signaling activates two major downstream signaling pathways: JAK2/STAT3 and Src homology‐2 domain‐containing phosphatase (SHP‐2)/extracellular signal‐regulated kinase (ERK). Among these pathways, STAT3 has been extensively studied as a critical mediator of chronic inflammation and immune dysregulation, and pharmacological inhibition of STAT3 has been proposed as a promising therapeutic strategy in inflammatory and autoimmune diseases (Ahmad et al. 2013). Each signaling pathway activates transcription factors and promotes gene expressions such as c‐fos, c‐jun, and nuclear factor IL‐6 (NF‐IL‐6) in SHP‐2/ERK, C‐reactive protein (CRP), IL‐1β, intracellular adhesion molecule 1 (ICAM‐1), and suppressor of cytokine signaling 3 (SOCS3) in JAK2/STAT3 (Lim et al. 2019; Page et al. 2005; Shi et al. 2000). Consequently, control of these genetic elements promotes to cell survival, differentiation and proliferation, and affects the inflammatory response, energy metabolism, and bone metabolism (Smith 2018). Thus, the regulation of IL‐6 signaling is an attractive strategy for treating inflammatory and bone metabolic diseases.

RA is a long‐term autoimmune disorder that impacts the joints (Fujimoto et al. 2008). The pain, stiffness, and swelling of joints, which are caused by hyperplasia of synovial tissue and the degradation of articular cartilage, bone, and ligaments, are representative symptoms of RA (Fujimoto et al. 2008; Scherer et al. 2020). The exact cause of RA remains unclear; however, various risk factors include age, sex, obesity, and family history (Scherer et al. 2020). The pathogenesis of RA is complex and mediated by various factors, including cytokines, proteolytic enzymes, and prostanoids (Guo et al. 2018). The CIA model is the most frequently utilized animal model for researching RA. Immunization with type II collagen (CII) leads to an autoimmune response against CII in the cartilage in the CIA model. As a T cell dependent disease, Th1 cells were considered major and pathogenic mediators of CIA (Lamacchia et al. 2010; Schulze‐Koops and Kalden 2001). However, interferon γ (IFN‐γ) and IL‐12p35, which are markers of Th1 cells, in knockout mice showed accelerated arthritis symptoms in the CIA model (Lee et al. 2013; Murphy et al. 2003). In addition, current research has shown that Th17 cells, which produce IL‐17, play a role in driving CIA development (Fujimoto et al. 2008). Th17 cells secrete TNF‐α, IL‐22, IL‐17, and granulocyte‐macrophage colony‐stimulating factor (GM‐CSF) (Hirota et al. 2018; Kikodze et al. 2016). These cells stimulate macrophages, synovial fibroblasts, osteoclasts, and endothelial cells (Wu et al. 2014). The stimulation of these cells contributes to angiogenesis, synovial tissue inflammation, and bone erosion in the joints within the CIA model. As naïve T cells differentiate into Th17 cells, IL‐6 activates the retinoic acid receptor‐related orphan nuclear receptor (RORγT), a key transcription factor essential for IL‐17 expression (Kimura and Kishimoto 2010). Therefore, blocking IL‐6 signaling reduces the polarization of Th17 cells and alleviates symptoms driven by Th17 cells (Nishihara et al. 2007). Recent studies further support the clinical relevance of the IL‐6/STAT3–Th17 axis in rheumatoid arthritis, highlighting this pathway as a key therapeutic target for disease modulation (Bakheet et al. 2020a).

*Portulaca oleracea* is a seasonal plant found extensively across Asia, Central Europe, Australia, the Middle East, and America (Uddin et al. 2014). This plant has been traditionally used for its antispasmodic, febrifuge, vermifuge, diuretic, and antiseptic properties (Hwang et al. 2016; Uddin et al. 2014). In a prior study, we isolated various feruloyl amides from *P. oleracea* , one of which was NHA (Uddin et al. 2014). NHA was first identified in the stem wood of *Hibiscus tiliaceus* (Chen et al. 2006). The biological activity of NHA has not been reported, except for its anti‐inflammatory activity (Hwang et al. 2016). To obtain a sufficient quantity of NHA, we synthesized NHA and identified a new compound as a by‐product: HAD.

In this research, we examined the impact of NHA and HAD on IL‐6‐induced STAT3 phosphorylation and their therapeutic potential in a CIA mouse model. The findings highlight the promise of these compounds as treatment options for RA, with their effects mediated by IL‐6.

## 2 Methadology

### 2.1 Materials and Cell Culture

Hep3B and U266 cells were maintained in DMEM and RPMI 1640 media, each supplemented with 10% fetal bovine serum, 2 mM glutamine, 100 U/mL penicillin, and 100 μg/mL streptomycin. The culture media, fetal bovine serum, and penicillin/streptomycin were sourced from Gibco BRL (Grand Island, NY, USA). Recombinant IL‐6 and TGF‐β were purchased from R&D Systems (Minneapolis, MN, USA). Antibodies against β‐actin and mouse were acquired from Sigma‐Aldrich (St. Louis, MO, USA), while FITC‐conjugated anti‐pSTAT3, CD4 antibodies, and Alexa Fluor 568‐conjugated anti‐IL‐17 antibody were supplied by Santa Cruz Biotechnology (Dallas, TX, USA). Anti‐CD3, anti‐CD28, anti‐IL‐2, anti‐IL‐4, and anti‐IFN‐γ antibodies were obtained from Invitrogen (Waltham, MA, USA), with additional antibodies sourced from Cell Signaling Technology (Boston, MA, USA). All other chemicals were procured from Sigma‐Aldrich.

### 2.2 Synthesis of NHA and HAD

A solution of 3, 5‐dimethoxy‐4‐hydroxyphenethylamine HCl (0.80 g, 3.4 mmol), ferulic acid (0.80 g, 4.1 mmol), and dimethylformamide (DMF, 24 mL) was prepared at room temperature. To this mixture, 1‐ethyl‐3‐(3‐dimethylaminopropyl) carbodiimide (EDC, 2.4 mL, 13.6 mmol) and triethanolamine (TEA, 1.8 mL) were added at −5°C and stirred for 10 min. The reaction mixture was then stirred at room temperature overnight. After removing the solvent under reduced pressure, the residue was purified using flash column chromatography on a silica gel column (SiO2, 120 g, CHCl3, 1:0 → 30:1, v/v), yielding 11 subfractions. Fraction 8 (868.2 mg) was further purified by reversed‐phase C18 flash column chromatography (C18, 130 g, H2O, 13:7 → 2:3, v/v), resulting in the isolation of NHA (168.5 mg) and its derivative HAD (52.7 mg), as confirmed by 1H, 13C, and 2D NMR (COSY, HMQC, HMBC, NOESY) and MS spectroscopic analysis. Detailed 1H, 13C, and MS spectroscopic data are provided in the Supporting Information.

### 2.3 Luciferase‐Based pSTAT3 Assay

Hep3B cells that stably express pSTAT3‐Luc were generated as previously described (Lim et al. 2019). The cells were plated in 96‐well plates at a density of 3 × 10^4^ cells per well. After 24 h, the cells were cultured in serum‐free DMEM for 12 h, followed by treatment with IL‐6 (10 ng/mL) or IL‐6 family cytokines (50 ng/mL) with or without NHA and HAD for an additional 12 h. Luciferase activity was quantified following the manufacturer's instructions (Promega Corp., Madison, WI, USA).

### 2.4 Cell Viability Assay

Hep3B cells were plated in 96‐well culture plates at a density of 3 × 10^4^ cells per well and incubated for 24 h. The cells were then treated with NHA and HAD at the specified concentrations for 24 h. Cell viability was assessed using an MTT assay, following the manufacturer's instructions (Sigma‐Aldrich).

### 2.5 Immunocytochemistry

Hep3B cells were plated on collagen‐coated coverslips in 6‐well plates and cultured overnight. The medium was replaced with serum‐free DMEM, and the cells were incubated for an additional 6 h. Then, the cells were treated with IL‐6 (10 ng/mL) for 30 min after being pre‐treated with 30 or 60 μM NHA and HAD for 1 h. Subsequently, the coverslips were rinsed with phosphate‐buffered saline (PBS) and fixed in ice‐cold methanol for 10 min. After fixation, the cells were blocked with 1% bovine serum albumin (BSA) in PBS containing 0.1% Tween 20 (PBST) for 30 min and incubated with FITC‐labeled anti‐pSTAT3 overnight. Finally, the coverslips were mounted and stained with DAPI using ProLong Gold Antifade Reagent with DAPI (Thermo Fisher Scientific, Waltham, MA, USA).

### 2.6 Western Blot Analysis

Whole‐cell or nuclear proteins were extracted using either cell lysis buffer (Cell Signaling Technology) or NE‐PER nuclear and cytoplasmic extraction reagents (Thermo Fisher Scientific). The proteins were then loaded onto 4%–12% SDS‐PAGE gels for separation. Following electrophoresis, the proteins were transferred to polyvinylidene fluoride (PVDF) membranes and blocked with tris‐buffered saline (TBS) containing 5% BSA. After blocking, the membrane was washed and incubated with the appropriate primary and secondary antibodies. Finally, protein bands were detected using the West‐Queen RTS Western Blot Detection Kit (iNtRON Bio, Seongnam, Korea). Details of the primary and secondary antibodies used for Western blot analysis, including sources and dilutions, are summarized in Table 1.

**TABLE 1:** Antibodies used for Western blot analysis.

Target	Molecular weight (kDa)	Antibody type	Source	Catalog number	Dilution
pSTAT3 (Tyr705)	86	Primary	Cell Signaling Technology (CST)	#9131 s	1:1000
STAT3	86	Primary	CST	#4409 s	1:1000
pERK1/2	44/42	Primary	CST	#9101 s	1:1000
ERK1/2	44/42	Primary	CST	#4348 s	1:1000
pJAK2	125	Primary	CST	#3771 s	1:1000
JAK2	125	Primary	CST	#3230 s	1:1000
β‐Actin	42	Primary	CST	#4967 s	1:1000
HRP‐conjugated anti‐rabbit IgG	—	Secondary	CST	#7074 s	1:2000

### 2.7 IL‐6/IL‐6 Receptor Binding Assay

IL‐6/IL‐6R binding affinity was measured using an IL‐6/IL‐6R biochemical homogeneous time‐resolved fluorescence (HTRF) assay kit (CisBio, Bedford, MA, USA) and modified solid ligand binding assay. IL‐6/IL‐6R biochemical homogeneous HTRF assay was performed according to the manufacturer's instructions. Briefly, 10, 30, and 60 μM NHA and HAD were dispensed into low volume 96 well white plates. Then, Tag1‐IL‐6 and Tag2‐IL‐6R were added. Next, the wells were treated with the anti‐Tag1 antibody conjugated with europium cryptate and the anti‐Tag2 antibody labeled with XL665 and incubated for 24 h at room temperature. HTRF signals were recorded by a Varioskan LUX multimode microplate reader (Thermo Fisher Scientific).

The IL‐6/IL‐6R binding assay, an adapted solid‐phase ligand binding technique, was conducted following the method outlined in a previous study (Lim et al. 2019). To summarize, IL‐6R (1 μg/mL) was applied to a 96‐well plate and allowed to incubate overnight at 4°C. Following PBS washing, the plate was blocked with 1% BSA in PBS for 1 h at room temperature. IL‐6 (50 ng/mL) was then added following a 1‐h pre‐treatment with 10, 30, and 60 μM NHA and HAD. Following IL‐6 treatment, an HRP‐conjugated anti‐IL‐6 antibody was added and incubated for 1 h at room temperature. The unbound antibody was then washed away with PBS. Next, the HRP substrate was introduced and incubated for 30 min. The reaction was then terminated by adding 1 M HCl. The optical density was recorded at 450 nm using a microplate reader.

### 2.8 Quantitative Real‐Time RT‐PCR

Total RNA was extracted using the PureLink RNA Mini Kit (Ambion, Foster City, CA, USA) following the manufacturer's instructions. Complementary DNA (cDNA) synthesis was carried out via reverse transcription using SuperScript III First Strand Synthesis SuperMix (Invitrogen). Real‐time PCR was conducted with the StepOnePlus Real‐Time PCR System (Applied Biosystems, Foster City, CA, USA) and TaqMan Gene Expression Master Mix (Applied Biosystems). Gene expression levels were normalized to human 18S rRNA, and relative gene expression was calculated using the 2^−ΔΔCt^ method. All primers were obtained from Applied Biosystems.

### 2.9 Animals

All experiments involving mice were conducted in accordance with relevant guidelines and regulations, including ethical standards for animal research, and were approved by the Institutional Animal Care and Use Committee of the Korea Food Research Institute (KRIBB‐AEC‐22266, KRIBB‐AEC‐23231). All methods are reported in accordance with the ARRIVE guidelines to ensure transparency and reproducibility in animal research. The euthanasia process was conducted in compliance with the ethical standards approved by the Institutional Animal Care and Use Committee. Mice were anesthetized using isoflurane to ensure minimal discomfort before euthanasia. Following anesthesia, euthanasia was performed by CO_2_ inhalation, adhering to the AVMA (American Veterinary Medical Association) Guidelines for the Euthanasia of Animals to ensure a humane and painless procedure.

### 2.10 Induction of CIA and Treatment With NHA and HAD

Five‐week‐old male DBA/1 mice were obtained from Orientbio (Seongnam, Korea). The mice were assigned to six groups and allowed to acclimate for 1 week. The number of animals per group was determined based on commonly used sample sizes in collagen‐induced arthritis models to ensure adequate statistical power while minimizing animal use. Mice were randomly assigned to experimental groups prior to treatment. Following the acclimation period, the mice received an injection of 100 μL of collagen emulsion, administered 2 cm from the base of the tail, for the initial immunization. Booster injections were administered 3 cm from the base of the tail 2 weeks after the first injection. For the initial immunization, type II chicken collagen was dissolved at 2 mg/mL in 0.05 M acetic acid and emulsified with complete Freund's adjuvant (CFA) containing 4 mg/mL of *Mycobacterium tuberculosis* (1:1, v/v). For the booster injection, type II chicken collagen was prepared at a concentration of 2 mg/mL in 0.05 M acetic acid and mixed with incomplete Freund's adjuvant (IFA) in a 1:1 (v/v) ratio. After the boost immunization, vehicle and 10 and 30 mg/kg NHA and HAD were injected intraperitoneally into the mice in each group every 2 days for 18 days. The doses of NHA and HAD (10 and 30 mg/kg) were chosen based on commonly used dose ranges for small‐molecule anti‐inflammatory compounds in murine models, with consideration of efficacy and tolerability. Following the experiment, the mice were euthanized, and their serum, knee joints, and spleens were harvested for subsequent analysis.

### 2.11 Assessment of Arthritis Severity

Arthritis severity was assessed based on the criteria outlined by (Fujimoto et al. 2008). In brief, the symptoms in each limb were visually inspected and rated on a scale from 0 to 4, with the following scores: 0 = no redness or swelling; 1 = mild redness and swelling of the ankle joint; 2 = mild redness and swelling affecting the whole paw; 3 = moderate redness and swelling involving the entire paw; and 4 = severe redness and swelling of the entire paw (Table S1).

### 2.12 ELISA Measurement of Serum IL‐17a and Anti‐CII IgG Levels

Blood samples were obtained through cardiac puncture following anesthesia induction. Serum was separated by centrifugation and stored at −80°C for subsequent analysis of anti‐CII IgG and IL‐17a. The concentrations of anti‐CII IgG and IL‐17a in the serum were determined using commercial ELISA kits (Chonderx, Woodinville, WA, USA) following the guidelines of the manufacturer.

### 2.13 Isolation of Naïve CD4 Positive T Cell and Th17 Cell Differentiation

The spleens from DBA/1 mice were collected and homogenized in PBS containing 2% FBS. The resulting cell suspension was filtered through a 70 μm nylon mesh strainer to remove any clumps and debris. Naive CD4+ T cells were isolated using the EasySep Mouse Naive CD4+ T Cell Isolation Kit (Stemcell Technologies, Vancouver, BC, Canada), following the provided protocol. For Th17 differentiation, the isolated cells were treated with NHA or HAD and stimulated with plate‐bound anti‐CD3 (5 μg/mL), anti‐CD28 (5 μg/mL), anti‐IL‐2 (10 μg/mL), anti‐IL‐4 (10 μg/mL), anti‐IFN‐γ (10 μg/mL), TGF‐β (2.5 ng/mL), and IL‐6 (25 ng/mL) for either 2 or 5 days.

### 2.14 Statistical Analysis

Statistical analysis was conducted on data obtained from triplicate experiments for in vitro studies and sextuplicate for in vivo studies. All quantitative data are expressed as means ± standard deviations (SD). The analyses were performed using Prism 5 software (GraphPad Software, San Diego, CA, USA), and statistical significance was assessed using one‐way ANOVA followed by Dunnett's test or an unpaired Student's *t*‐test.

## 3 Results

### 3.1 NHA and HAD Inhibit IL‐6‐Induced Luciferase Activity in pSTAT3‐Luc‐Expressing Hep3B Cells

**FIGURE 1:**
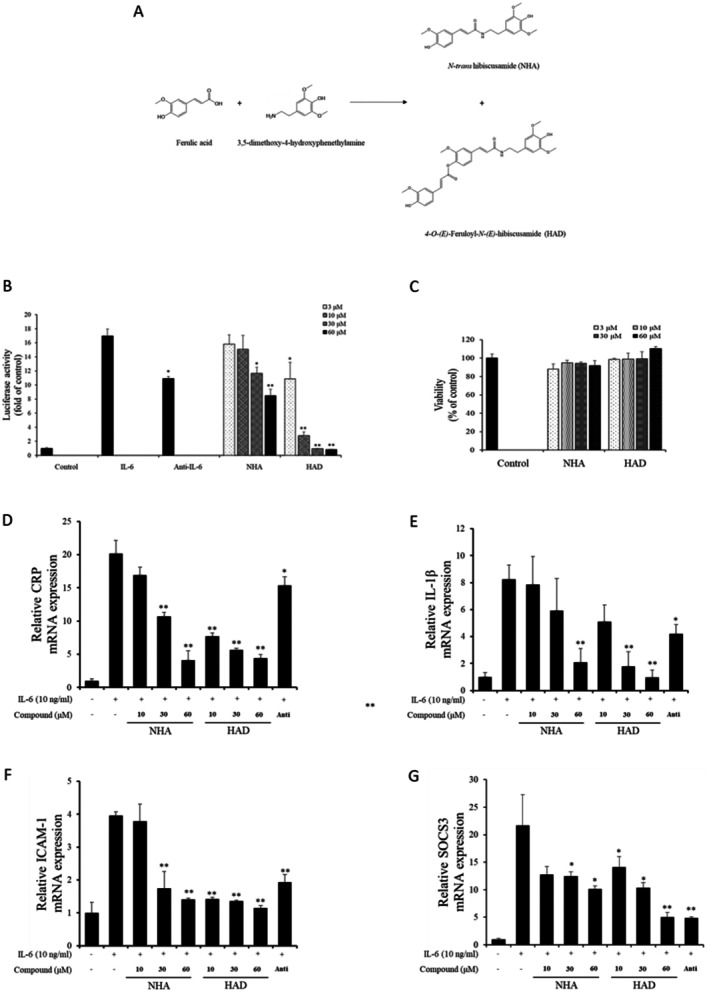
Inhibition of pSTAT3‐induced luciferase activity and IL‐6‐mediated gene expression by NHA and HAD. (a) Overview of the synthesis process for NHA and HAD. (b) pSTAT3‐luciferase‐expressing Hep3B cells were plated in 96‐well plates and treated with the indicated concentrations of NHA and HAD for 1 h prior to exposure to IL‐6 (10 ng/mL) for 12 h. (c) Hep3B cells were seeded in 96‐well plates and treated with the specified concentrations of NHA and HAD for 24 h, followed by the MTT assay. (d‐g) Hep3B cells were seeded in 6‐well plates, pretreated with varying concentrations of NHA and HAD for 1 h, and then stimulated with IL‐6 (10 ng/mL) for 6 h. After incubation, cells were collected, and gene expression levels of (d) CRP, (e) IL‐1β, (f) ICAM‐1, and (g) SOCS3 were assessed using quantitative real‐time RT‐PCR. The results are presented as the mean ± SD (*n* = 3) of three individual experiments. The statistical significance was assessed using one‐way ANOVA followed by an unpaired Student's *t*‐test. *p < 0.05, **p < 0.01 compared with the IL‐6‐treated group. Anti, 200 ng/mL anti‐IL‐6 neutralizing antibody.

The IL‐6/STAT3 pathway plays a crucial role in the progression of various cancers and inflammatory disorders. Thus, inhibiting this signaling pathway is an option when developing therapeutic agents. To investigate the inhibitory effect of NHA and HAD (Figure 1a) on IL‐6/STAT3 signaling, a pSTAT3‐induced luciferase assay was performed. Both NHA (30 and 60 μM) and HAD (3, 10, 30 and 60 μM) significantly inhibited pSTAT3‐induced luciferase activity (Figure 1b). An anti‐IL‐6 neutralizing antibody (200 ng/mL) was used as a positive control. An MTT assay was conducted to assess the cytotoxicity of NHA and HAD, and the results indicated that neither compound exhibited cytotoxic effects (Figure 1c).

### 3.2 Inhibition of IL‐6‐Induced Gene Expression by NHA and HAD

As a transcription factor, the phosphorylated STAT3 dimer regulates various genes, including those associated with proliferation, inflammation, and migration. To confirm that NHA and HAD inhibit STAT3‐inducible gene expression, qRT‐PCR analysis of IL‐1β, CRP, SOCS3, and ICAM‐1 was performed. The expression of these genes was downregulated by NHA and HAD in accordance with the dose (Figure 1d–g). In particular, 60 μM NHA and 30 and 60 μM HAD significantly inhibited the expression of these genes.

### 3.3 NHA and HAD Inhibit the Nuclear Translocation of pSTAT3

**FIGURE 2:**
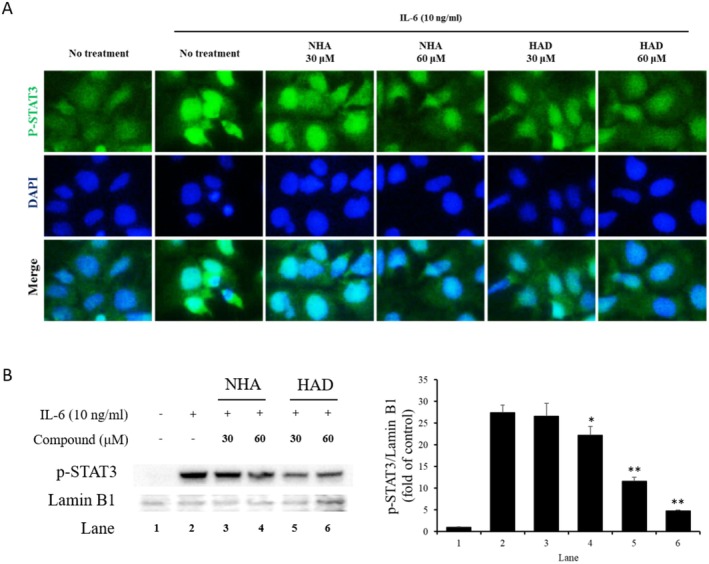
Effect of NHA and HAD on the nuclear translocation of pSTAT3. (a) Hep3B cells were cultured on collagen‐coated coverslips in 6‐well plates. After a 1‐h pretreatment with NHA and HAD, the cells were exposed to IL‐6 (10 ng/mL) for 30 min. Following this, the cells were fixed and stained with Alexa 488‐conjugated anti‐pSTAT3 antibodies, along with DAPI for nuclear visualization. DAPI was used to highlight the nucleus. (b, c) Hep3B cells were seeded in 6‐well plates and pretreated with NHA and HAD for 1 h. Subsequently, the cells were stimulated with IL‐6 (10 ng/mL) for 30 min, and nuclear proteins were isolated. (b) The extracted proteins were analyzed using Western blotting. (c) The optical density of the bands was quantified with ImageJ software. The results are presented as the mean ± SD (*n* = 3) of three individual experiments. The statistical significance was assessed using one‐way ANOVA followed by an unpaired Student's *t*‐test. *p < 0.05, **p < 0.01 compared with the IL‐6‐treated group.

The phosphorylated STAT3 dimer complex is formed in the cytosol and translocated into the nucleus. Then, the complex promotes STAT3‐related gene expression. To confirm the downregulation of STAT3‐inducible gene expression caused by the translocation of pSTAT3, immunofluorescence staining and Western blot analysis were performed. In the immunofluorescence staining result, IL‐6 treatment increased the nuclear translocation and expression of total pSTAT3, but they were decreased by treatment with NHA and HAD (Figure 2a). The decreased nuclear translocation of pSTAT3 by NHA and HAD was confirmed by Western blot analysis (Figure 2b,c).

### 3.4 NHA and HAD Inhibit IL‐6/STAT3 Signaling Pathway Components

**FIGURE 3:**
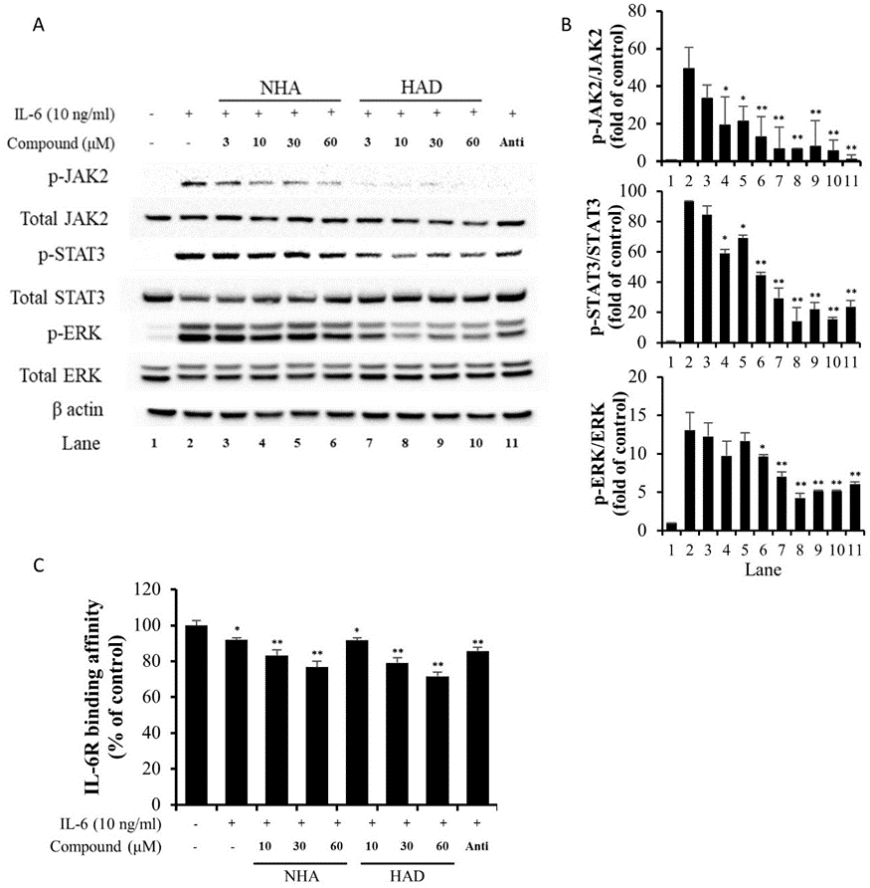
Inhibitory effects of NHA and HAD on IL‐6 signaling molecules and IL‐6/IL‐6R binding. (a, b) U266 cells were cultured in 6‐well plates and pretreated with the specified concentrations of NHA and HAD for a certain duration. Following this, the cells were exposed to IL‐6 (10 ng/mL) for 20 min. After incubation, proteins were extracted. (a) Phosphorylation of IL‐6 signaling molecules was analyzed by Western blot analysis. (b) The optical densities of the bands were quantified using ImageJ software. (c) The indicated concentrations of NHA and HAD were added to low‐volume 96‐well white plates. Then, Tag1‐IL‐6 and Tag2‐IL‐6R were dispensed. Next, the wells were treated with homogeneous time‐resolved fluorescence (HTRF) substrate‐labeled anti‐Tag1 antibodies and anti‐Tag2 antibodies and incubated for 24 h at room temperature. The HTRF signal was measured using a microplate reader, and the results are presented as the mean ± SD (*n* = 3) from three independent experiments. The statistical significance was assessed using one‐way ANOVA followed by an unpaired Student's *t*‐test. *p < 0.05, **p < 0.01 compared with the IL‐6‐treated group. Anti, 200 ng/mL anti‐IL‐6 neutralizing antibody.

To explore the underlying molecular mechanism of NHA and HAD, Western blot analysis was performed to evaluate the phosphorylation levels of key signaling components in the IL‐6 pathway (Figure 3a). U266 cells were employed in place of Hep3B cells, as IL‐6‐induced JAK2 phosphorylation could not be detected in Hep3B cells (Hwang et al. 2016). NHA and HAD effectively reduced the IL‐6‐induced phosphorylation of STAT3 and ERK, key downstream signaling components of the IL‐6 pathway. Additionally, treatment with NHA and HAD significantly inhibited the phosphorylation of JAK2, an upstream regulator of STAT3 and ERK activation (Figure 3a,b).

### 3.5 NHA and HAD Influence the Interaction Between IL‐6 and IL‐6R

Previous results demonstrated that NHA and HAD suppressed the IL‐6‐triggered phosphorylation of signaling molecules. To determine the molecular mechanism by which NHA and HAD affect IL‐6 signaling, whether NHA and HAD inhibit phosphorylation of signaling molecules or IL‐6 and IL‐6R binding affinity was investigated. Thus, IL‐6 and IL‐6R binding assays were performed. The findings indicated that NHA and HAD reduced the binding affinity between IL‐6 and IL‐6R (Figure 3c and Figure S1).

### 3.6 NHA and HAD Reduce Disease Severity and Prevent Cartilage Damage in CIA Mice

**FIGURE 4:**
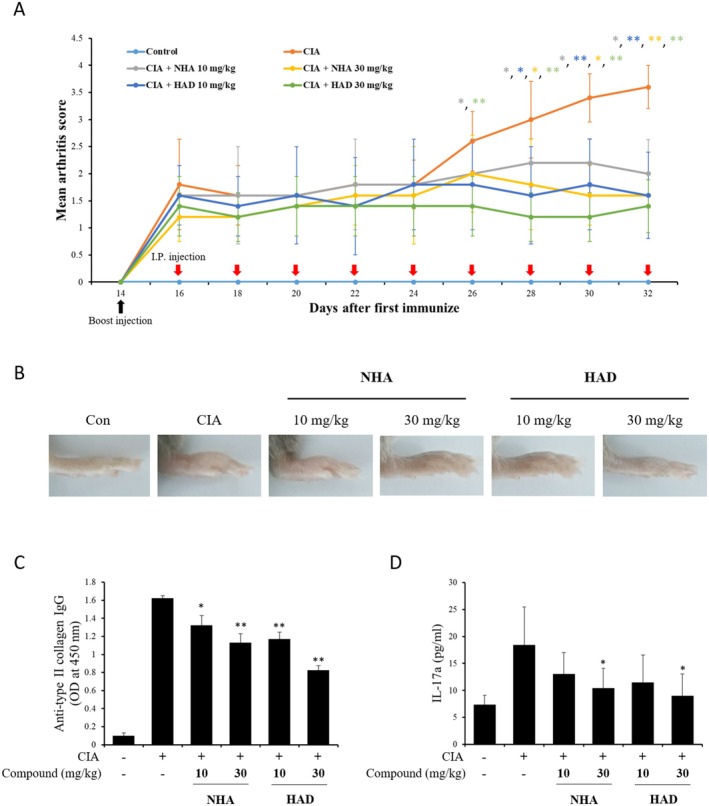
The effect of NHA and HAD on the CIA mouse model. (a) Arthritis scores of CIA mice treated with NHA and HAD. (b) Representative images of paws from NHA‐ and HAD‐treated CIA mice. (c, d) Serum concentrations of (c) anti‐CII IgG and (d) IL‐17a in CIA mice treated with NHA and HAD. The values are expressed as the means ± SD (*n* = 6 mice per group). The statistical significance was assessed using one‐way ANOVA followed by Dunnett's test. *p < 0.05, **p < 0.01 compared with the CIA group.

To examine whether NHA‐ and HAD‐induced IL‐6/IL‐6R binding inhibition could reduce the symptoms of RA, CIA mice were treated with NHA and HAD. Following booster immunizations, NHA and HAD were administered to the mice via intra‐peritoneal injection at doses of 10 and 30 mg/kg every other day for a duration of 18 days. Treatment with NHA and HAD dramatically decreased the severity of CIA (Figure 4a,b). The development of arthritis was linked to the serum levels of anti‐CII antibodies. Treatment with NHA and HAD markedly decreased the levels of anti‐CII IgG and IL‐17a compared to those observed in the CIA group (Figure 4c,d).

### 3.7 NHA and HAD Downregulate the Expression of Th17‐Specific Genes in CIA Mice

**FIGURE 5:**
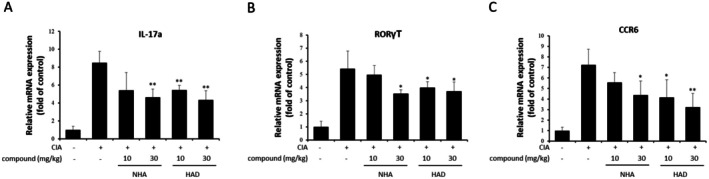
The effect of NHA and HAD on Th17‐specific gene expression in the CIA mouse model. Total mRNA was isolated from the spleens of CIA mice treated with NHA and HAD and subjected to qPCR analysis. The expression levels of (a) IL‐17a, (b) RORγT, and (c) CCR6 were assessed. The data are expressed as the means ± SD (*n* = 6 mice per group). The statistical significance was assessed using one‐way ANOVA followed by Dunnett's test. *p < 0.05, **p < 0.01 compared with the CIA group.

Th17 cells are major mediators of CIA progression. During the Th17 cell differentiation process, STAT3 plays a critical role in activating RORγT, which is a transcriptional regulator related to IL‐17 expression. To investigate how NHA and HAD affect Th17 cells, the expression of Th17 marker genes, including IL‐17a, RORγT, and chemokine receptor 6 (CCR6), in the spleens of NHA‐ and HAD‐treated CIA mice was analyzed by qRT‐PCR. Both NHA and HAD downregulated Th17 marker genes; however, 10 mg/kg NHA did not significantly downregulate Th17 marker genes (Figure 5a–c).

### 3.8 NHA and HAD Inhibit Phosphorylation of STAT3 During the In Vitro Differentiation of Th17 Cells

**FIGURE 6:**
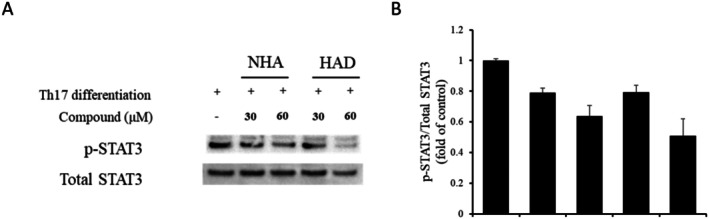
The effect of NHA and HAD on in vitro Th17 cell differentiation. Naive CD4+ T cells were treated with NHA and HAD, and after 2 days of stimulation, the cells were harvested for protein extraction. (a) The levels of pSTAT3 and total STAT3 were assessed through Western blot analysis, and (b) the optical densities of the bands were quantified using ImageJ software. The data are presented as the means ± SD (*n* = 3). The statistical significance was assessed using one‐way ANOVA followed by an unpaired Student's *t*‐test. *p < 0.05, **p < 0.01 compared with the only Th17 differentiation stimulated group.

Earlier findings indicated that NHA and HAD inhibit IL‐6‐induced STAT3 phosphorylation and suppress Th17 cell differentiation. However, their specific impact on STAT3 phosphorylation during Th17 cell differentiation had not been clarified. To investigate this, Western blot analysis was conducted. The results demonstrated that treatment with NHA and HAD markedly reduced STAT3 phosphorylation (Figure 6a,b).

## 4 Discussion

This study aimed to investigate the inhibitory effects of N‐trans‐hibiscusamide (NHA) and its derivative on interleukin‐6 (IL‐6)/STAT3 signaling and to evaluate their therapeutic potential in a collagen‐induced arthritis (CIA) model. We demonstrate that NHA and HAD suppress IL‐6–induced STAT3 activation, inhibit transcriptional regulation of pro‐inflammatory genes, and attenuate Th17 cell differentiation both in vitro and in vivo. Furthermore, these compounds significantly alleviated disease severity and autoimmune responses in the CIA mouse model, highlighting their potential as modulators of IL‐6/STAT3–driven inflammatory pathways. IL‐6 is a versatile cytokine that influences and controls the immune response, energy balance, and bone metabolism (Smith 2018). Due to its pleiotropic properties, the regulation of IL‐6 signaling is considered a strategy for developing therapeutic agents to treat numerous diseases, such as Crohn's disease, inflammatory bowel disease, RA, osteoporosis and various cancers (Smith 2018; Wang and Sun 2014). AG490, which is a JAK2 inhibitor, has been investigated as a therapeutic agent for pancreatic cancer, ovarian cancer, inflammatory fever, fibrosis and autoimmune diseases (Gasparitsch et al. 2019; Kobayashi et al. 2015; Park et al. 2014). Stattic, which is a STAT3 inhibitor, has been studied as an antitumor, anti‐osteoporosis and osteoarthritis agent (Latourte et al. 2017; Li et al. 2018; Pan et al. 2013). Thus, discovering an IL‐6/STAT3 blocking agent is important for developing therapeutic agents against these diseases. Recent studies have emphasized the therapeutic relevance of targeting IL‐6/STAT3 signaling in inflammatory and autoimmune diseases, particularly through modulation of immune cell differentiation and inflammatory gene expression (Ansari et al. 2021; Bakheet et al. 2020a). While these studies have established the importance of IL‐6/STAT3 inhibition, the identification of novel small‐molecule or natural compound–derived inhibitors with efficacy in experimental arthritis models remains limited.

In this study, NHA and HAD showed potent inhibitory effects on IL‐6 signaling. Both compounds downregulated IL‐6‐induced luciferase activity in pSTAT3‐Luc cells. To confirm their inhibitory effects on IL‐6‐induced genes such as CRP, SOCS3, ICAM‐1, and IL‐1β, we performed qRT‐PCR analysis and the PCR results indicated that these genes were downregulated by the compounds treatment. There are two major regulatory mechanisms associated with gene expression: transcriptional and posttranscriptional regulation. As a transcription factor, STAT3 binds its binding region and induces the expression of genes (Liu et al. 2008). After transcription, RNA binding proteins or microRNAs decrease RNA stability and breakdown mRNA as a form of posttranscriptional regulation (Bevilacqua et al. 2003). The immunocytochemistry results confirmed that NHA and HAD affected the nuclear translocation of pSTAT3. The PCR and immunocytochemistry results indicated that NHA and HAD did not affect posttranscriptional regulation; however, they inhibited transcription factors.

Various compounds have been identified that block IL‐6 signaling via distinct mechanisms. For example, *20‐S,21‐*epoxy‐resibufogenin‐*3*‐formate (ERBF), *20‐S,21‐*epoxy‐resibufogenin‐*3*‐acetate (ERBA), and LMT‐28 target IL‐6R and gp130 (Heo et al. 2016). AG490 directly inhibits JAK2, and piperlongumine downregulates STAT3 nuclear translocation (Kobayashi et al. 2015; Park et al. 2014). IL‐6 signaling is involved in various biological responses, and it is important to know how compounds exert their effects. In a study investigating the molecular mechanisms, NHA and HAD were found to inhibit IL‐6‐induced activation of STAT3, ERK, and JAK2, which are key components in the downstream signaling of IL‐6. These compounds also inhibited IL‐6 and IL‐6R binding affinity. This result indicates that NHA and HAD inhibit IL‐6 signaling by regulating IL‐6 and IL‐6R binding. However, to verify the inhibitory effect on IL‐6/IL‐6R binding, additional experimental results are needed. For example, IL‐6 or IL‐6R binding affinities and binding sites should be investigated to determine the exact inhibitory mechanisms. We also investigated how NHA and HAD influence STAT3 activation triggered by IL‐6 family cytokines in Hep3B cells that express pSTAT3‐luciferase. The findings demonstrated that NHA did not reduce luciferase activity, while HAD significantly inhibited it (Figure S2). These findings suggest that HAD may directly regulate IL‐6 signaling molecules or interfere with the binding of IL‐6 family cytokines to their receptors, as well as the dimerization of IL‐6 family cytokine receptors with gp130.

RA is a persistent autoimmune condition (Fujimoto et al. 2008). In the development of RA, CD4+ T cells, particularly IL‐17‐producing Th17 cells, play crucial roles (Fujimoto et al. 2008; Guo et al. 2018; Scherer et al. 2020). During the development of Th17 cells, the IL‐6/STAT3 axis plays a crucial role and activates RORγT (Capone and Volpe 2020; Nishihara et al. 2007). Harris et al. demonstrated that STAT3 signaling was a key factor in regulating Th17 differentiation during the early stage of experimental autoimmune encephalomyelitis (EAE) in STAT3‐knockout mice (Harris et al. 2007). In that experiment, endogenous Th17 cells were absent in STAT3‐knockout mice. Furthermore, researchers have shown that inhibiting IL‐6/STAT3 downregulates Th17 differentiation and alleviates CIA symptoms (Fujimoto et al. 2008; Nishihara et al. 2007). In line with these previous findings, our results further extend current knowledge by demonstrating that NHA and HAD suppress Th17‐associated immune responses through transcriptional inhibition of STAT3 signaling, thereby providing functional and mechanistic evidence for IL‐6/STAT3 blockade in experimental arthritis (Ansari et al. 2021; Bakheet et al. 2020b).

Despite these findings, several limitations of the present study should be acknowledged. First, although NHA and HAD were shown to inhibit IL‐6/STAT3 signaling and reduce IL‐6/IL‐6R binding affinity, the precise molecular interactions, including direct binding sites and affinities, were not fully characterized and warrant further investigation. Second, this study focused on pharmacological efficacy in vitro and in a collagen‐induced arthritis mouse model; therefore, comprehensive pharmacokinetic, toxicity, and long‐term safety evaluations were not performed. Third, the therapeutic effects were assessed using a single experimental arthritis model, and additional disease models may be required to further validate the generalizability of these findings. Addressing these limitations in future studies will be essential to fully elucidate the therapeutic potential of NHA and HAD. This study explored the impact of NHA and HAD in a CIA mouse model. It is thought that these compounds could moderate CIA through the regulation of Th17 differentiation due to their ability to block IL‐6 signaling. The results showed that NHA and HAD reduced the severity of CIA, such as swelling, erythema, and abnormal joint function. It has been reported that blocking STAT3 also decreases RANKL expression in osteoblasts, thereby mitigating joint damage caused by osteoclasts (Latourte et al. 2017; Wang et al. 2021). Thus, recent studies have focused on IL‐6/STAT3 signaling inhibitors to develop therapeutic agents for RA. NHA and HAD decreased serum levels of IL‐17a and anti‐CII IgG compared with the CIA group. The concentration of anti‐CII IgG is associated with the intensity of CIA symptoms and reflects the autoimmune activity of B cells (Bäcklund et al. 2003). NHA and HAD also downregulated Th17‐specific genes such as IL‐17a, CCR6, and RORγT in the spleens of CIA mice. These genes play a crucial role in the differentiation of Th17 cells. Additionally, NHA and HAD reduced Th17 cell differentiation and inhibited STAT3 phosphorylation during the in vitro differentiation of Th17 cells. These results demonstrate that NHA and HAD could be possible candidates for a therapeutic agent of Th17 cell mediated diseases. Although no clinical studies have yet been reported for NHA or HAD, the present findings provide preclinical evidence supporting their potential as lead compounds targeting IL‐6/STAT3‐mediated inflammatory pathways relevant to rheumatoid arthritis.

## 5 Conclusion

In summary, our research underscores the therapeutic potential of NHA and HAD in alleviating diseases driven by IL‐6 signaling, especially rheumatoid arthritis and other conditions involving Th17 cells. Notably, this study is the first to demonstrate the efficacy of N‐trans‐hibiscusamide and its derivative in a collagen‐induced arthritis model through modulation of the IL‐6/STAT3–Th17 axis. We clarified the inhibitory effects of NHA and HAD on IL‐6‐induced signaling pathways, such as the modification of Th17 cell differentiation and the downregulation of STAT3 phosphorylation through a series of experiments. In a murine model of CIA, NHA and HAD demonstrated notable efficacy in reducing disease severity, joint inflammation, and autoimmune responses. These findings distinguish NHA and HAD from previously reported IL‐6/STAT3 inhibitors and highlight their potential as novel natural compound–derived therapeutic candidates. Furthermore, our results highlight the significance of RA treatment options that target the IL‐6/STAT3 axis in relation to other autoimmune illnesses. Further investigation into the precise molecular mechanisms underlying the inhibitory effects of NHA and HAD on IL‐6 signaling pathways is warranted to advance their development as promising therapeutic agents for clinical applications.

## Author Contributions

H.J.L., S.G.B., and J.B. contributed equally to this work and share first authorship. H.J.L. was primarily responsible for conceptualization, data analysis, and manuscript drafting, while S.G.B. and J.B. focused on experimental design, data acquisition, and visualization. Y‐.S.W., S.H.C., and S‐.J.L. supervised the research, provided critical insights, and finalized the manuscript. N.C., E.J.P., and S.W.L. contributed to methodology development and data interpretation, M.K. and S.H‐.L. assisted with statistical analyses and technical support. All authors reviewed and approved the final manuscript.

## Funding

This research did not receive any specific grant from funding agencies in the public, commercial, or not ‐for‐profit sectors.

## Conflicts of Interest

The authors declare no conflicts of interest.

## Supporting information


**Data S1:** fsn371601‐sup‐0001‐Supinfo.pdf.

## Data Availability

All data generated or analyzed during this study are included in this published article and its Supporting Information files. Additional datasets used and/or analyzed during the study are available from the corresponding author upon reasonable request.
