# Supplementary material for: Inhibition of IL‐6/STAT3 Signaling by N‐Trans‐Hibiscusamide and Its Derivative in a Mouse Model of Collagen‐Induced Arthritis
Source: Food Sci Nutr. 2026 Mar 4;14(3):e71601. doi: 10.1002/fsn3.71601 (PMC12960055; doi:10.1002/fsn3.71601)
Supplement: Supplementary file 1 — Data S1: fsn371601‐sup‐0001‐Supinfo.pdf. [file FSN3-14-e71601-s001.pdf]

The 1H, 13C, and MS spectroscopic data of NHA and HAD

NHA (1)

Yield 11%; White solid; ESI-MS: m/z 374 [M+H]+; 1H NMR (600 MHz, methanol-d4) δH: 7.44 (1H, d, J = 15.6 Hz, H-7), 7.10 (1H, d, J = 1.8, H-2), 7.02 (1H, dd, J = 8.4, 1.8 Hz, H-6), 6.79 (1H, d, J = 8.4, H-5), 6.52 (2H, s, H-2′, 6′), 6.41 (1H, d, J = 15.6 Hz, H-8), 3.50 (1H, t, J = 7.2 Hz, H-8′) 2.77 (1H, t, J = 7.2 Hz, H-7′), 3.88 (3H, s, 3-OCH3), 3.82 (6H, s, 3′, 5′-OCH3); 13C NMR (150 MHz, methanol-d4) δC: 169.3 (C-9), 150.0 (C-4′), 149.4 (C-3), 149.3 (C-3′, 5′), 142.2 (C-7), 135.2 (C-4′), 131.4 (C-1), 128.4 (C-1′), 123.3 (C-6), 118.9 (C-8), 116.6 (C-5), 111.7 (C-2), 107.2 (C-2′, 6′), 42.6 (C-8′), 36.8 (C-7′), 56.9 (3′, 5′-OCH3), 48.5 (3-OCH3).

HAD (2)

Yield 3%; White solid; ESI-MS: m/z 550 [M+H]+; 1H NMR (600 MHz, methanol-d4) δH: 7.78 (1H, d, J = 16.2 Hz, H-7″), 7.52 (1H, d, J = 15.6 Hz, H-7), 7.26 (2H, m, H-2, 2″, overlap), 7.18 (1H, dd, J = 7.8, 1.8 Hz, H-6), 7.15 (1H, dd, J = 8.4, 1.8 Hz, H-6″), 7.10 (1H, d, J = 8.4 Hz, H-5″), 6.84 (1H, d, J = 8.4 Hz, H-5″), 6.58 (1H, d, J = 15.6 Hz, H-8), 6.58 (1H, d, J = 16.2 Hz, H-8″), 6.53 (2H, s, H-2′, 6′) 3.52 (1H, t, J = 7.8 Hz, H-8′), 2.79 (1H, t, J = 7.8 Hz, H-7′), 3.91 (3H, s, 3″-OCH3), 3.86 (3H, s, 3-OCH3), 3.83 (6H, s, 3′, 5′-OCH3); 13C NMR (150 MHz, methanol-d4) δC: 168.6 (C-9), 167.2 (C-9″), 153.3 (C-3), 151.2 (C-4″), 149.6 (C-3″), 149.4 (C-3′, 5′), 148.9 (C-7″), 142.7 (C-4), 141.2 (C-7), 135.4 (C-1), 135.2, (C-4′), 131.3 (C-1′), 127.7 (C-1″), 124.7 (C-6″), 124.5 (C-5), 122.3 (C-8), 121.7 (C-6), 116.7 (C-5″), 114.3 (C-8″), 112.7 (C-2), 112.1 (C-2″), 107.2 (C-2′, 6″), 42.6 (C-8′), 36.8 (C-7′), 56.9 (3′, -5′-OCH3), 56.6 (3″-OCH3), 56.5 (3-OCH3); HRESI-TOF-MS: m/z 548.1909 [M−H]− (calcd for C30H32NO9, 548.1926).

Supplementary Table S1. The arthritis severity score

Arthritis score	Symptom
0	No erythema or swelling
1	Erythema or mild swelling of the ankle joint
2	Mild erythema or mild swelling involving the entire paw
3	Erythema and moderate swelling involving the entire paw
4	Erythema and sever swelling involving the entire paw

Supplementary Figure S1

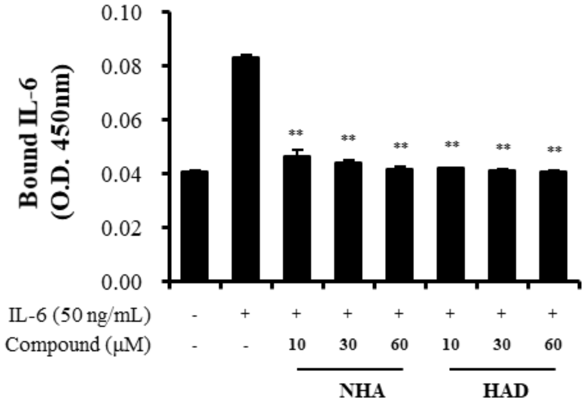

Supplementary Figure S1. ELISA for IL-6 Binding in the Presence of NHA and HAD Pre-treatment. A 96-well microplate was coated with IL-6R and subsequently blocked with 1% BSA in PBS. IL-6 was then added for 1 hour following a 1-hour pre-treatment with NHA and HAD. After IL-6 treatment, unbound IL-6 was washed out using PBS. The wells were then incubated with an HRP-conjugated anti-IL-6 antibody for 1 hour. Following incubation, the plate was washed with PBS, and HRP substrate was added for 30 minutes. The reaction was stopped by adding 1M HCl.

Supplementary Figure S2

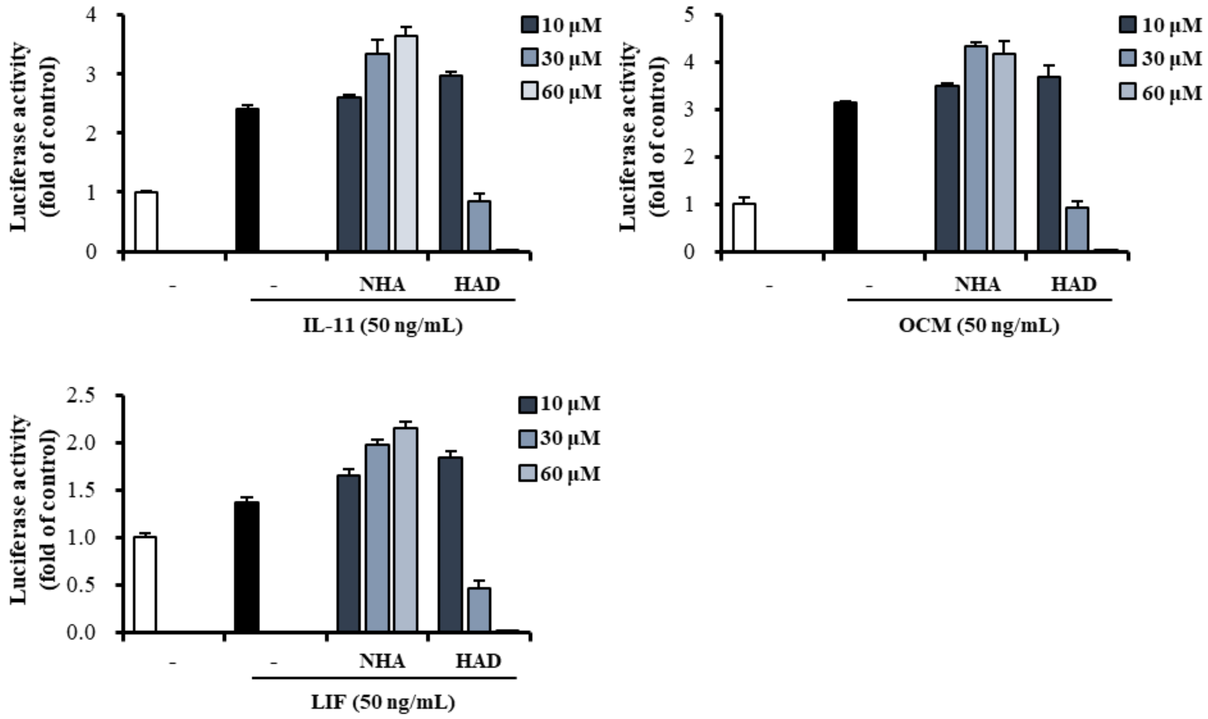

Supplementary Figure S2. Inhibition of STAT3 Activation by NHA and HAD in Hep3B Cells Pre-treated with IL-6 Family Cytokines. Hep3B cells expressing pSTAT3-luciferase were seeded in 96-well plates and pre-treated with the indicated concentrations of NHA and HAD for 1 hour. Following pre-treatment, cells were exposed to IL-6 family cytokines (50 ng/ml) for 12 hours to assess the effects of NHA and HAD on STAT3 activation.

Supplementary Figure S3

Uncropped western blotting images

**p-STAT3 (Figure 2B)**	**SREBP-1 (Figure 2B)**
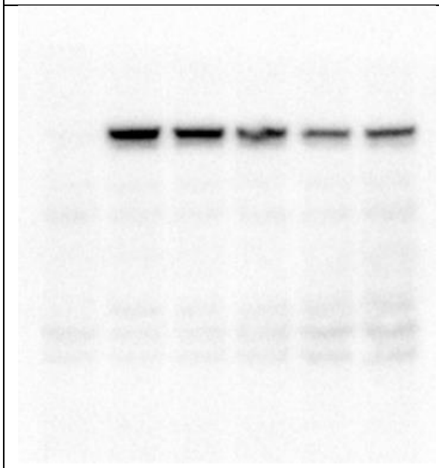	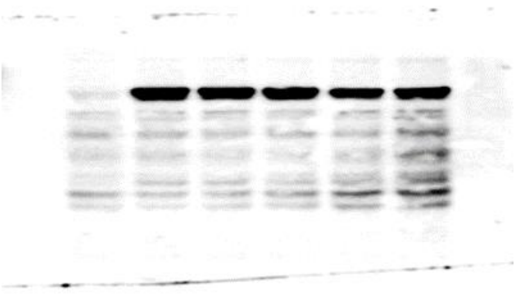
**p-JAK2 (Figure 3A)**	**Total JAK2 (Figure 3A)**
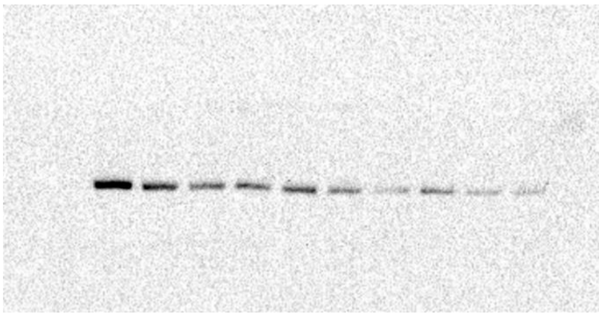	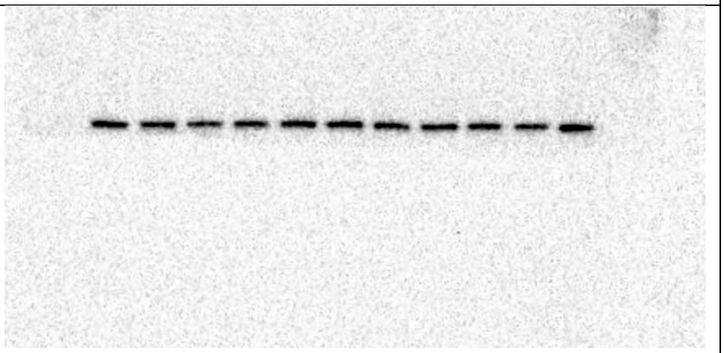
**p-STAT3 (Figure 3A)**	**Total STAT3 (Figure 3A)**
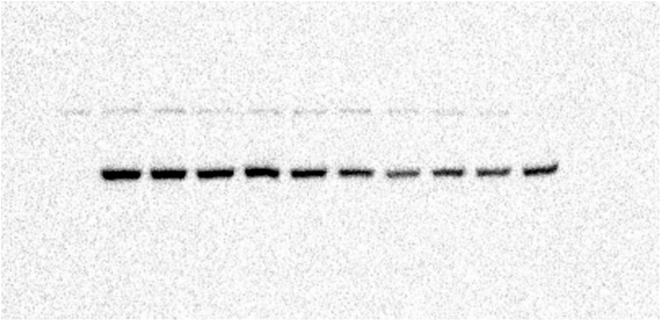	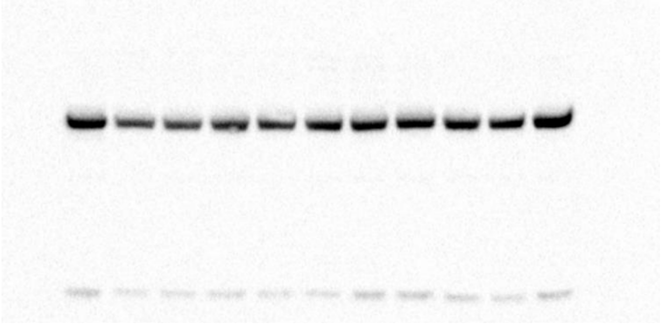

**p-ERK (Figure 3A)**	**Total ERK (Figure 3A)**
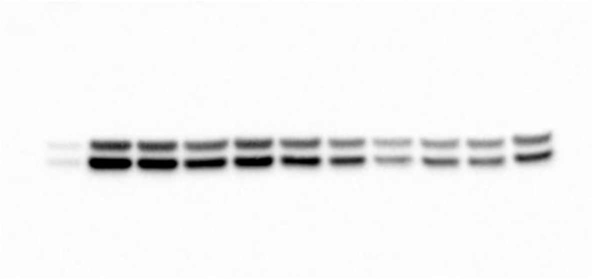	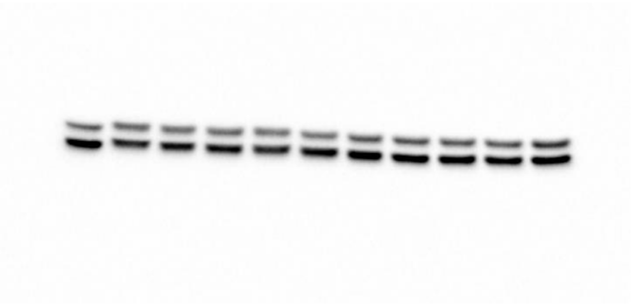
**β actin (Figure 3A)**	**p-STAT3 (Figure 6A)**
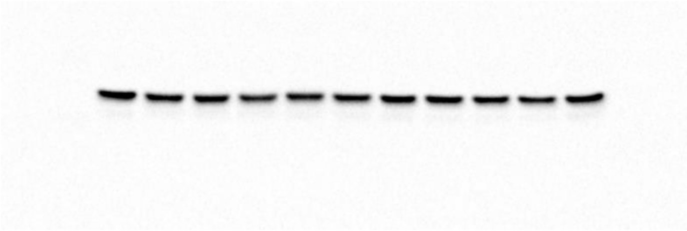	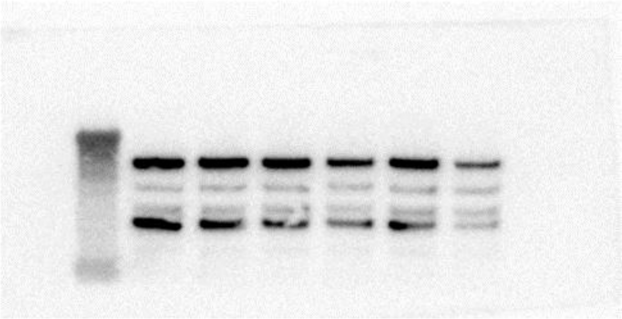
**Total STAT3 (Figure 6A)**	
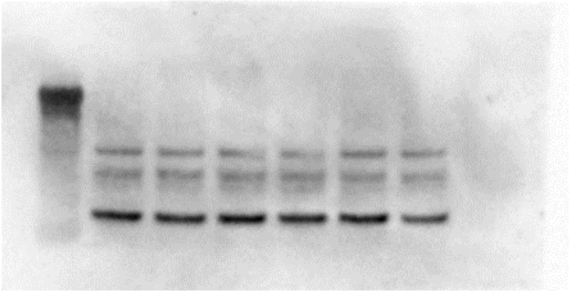	

Supplementary Figure S3. Uncropped Western Blot Images. The full, uncropped western blot images corresponding to the main figures are shown. The blots include p-STAT3, SREBP-1, p-JAK2, total JAK2, total STAT3, p-ERK, total ERK, and β-actin.

Supplementary Figure S4

**p-ERK**	**p-ERK-1**
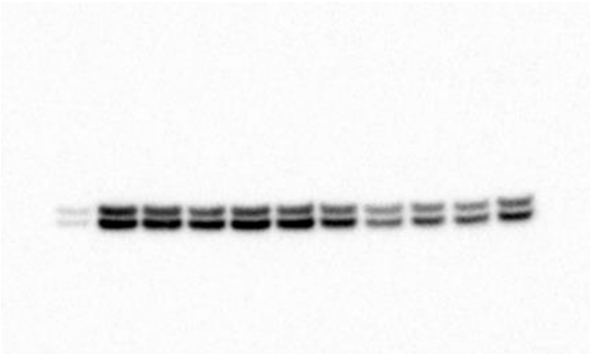	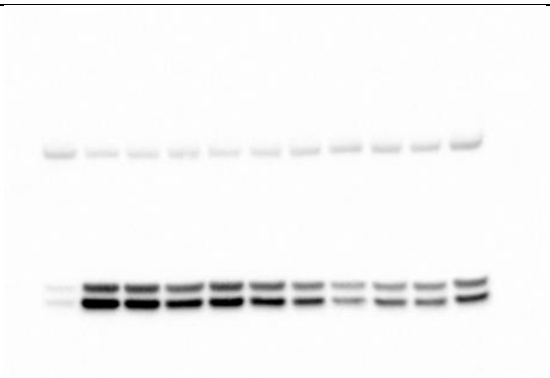
**Total ERK**	
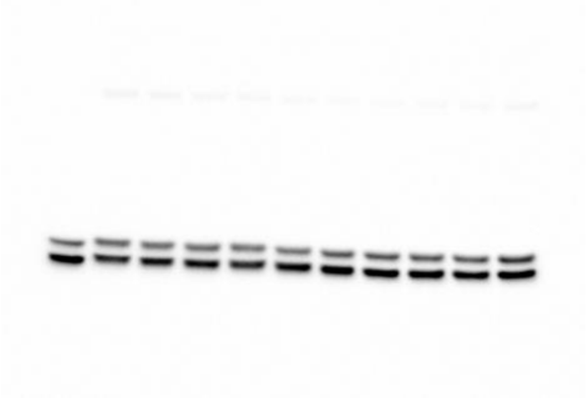	

Supplementary Figure S4. Multiple Exposure Images of p-ERK and Total ERK Western Blots. Two uncropped western blot images of p-ERK (captured using manual exposure mode at different exposure times between 2–10 seconds) and one image of total ERK (captured using auto exposure mode) corresponding to Figure 3A.
